# Silver nanoparticles-containing dual-function hydrogels based on a guar gum-sodium borohydride system

**DOI:** 10.1038/srep36497

**Published:** 2016-11-07

**Authors:** Lei Dai, Ben Nadeau, Xingye An, Dong Cheng, Zhu Long, Yonghao Ni

**Affiliations:** 1 Department of Chemical Engineering, University of New Brunswick, Fredericton, NB E3B 5A3, Canada; 2 Key Laboratory of Eco-textiles, Ministry of Education, Jiangnan University, Wuxi 214122, China

## Abstract

Dual-function hydrogels, possessing both stimuli-responsive and self-healing properties, have recently attracted attention of both chemists and materials scientists. Here we report a new paradigm using natural polymer (guar gum, GG) and sodium borohydride (NaBH_4_), for the preparation of silver nanoparticles (AgNPs)-containing smart hydrogels in a simple, fast and economical way. NaBH_4_ performs as a reducing agent for AgNPs synthesis using silver nitrate (AgNO_3_) as the precursor. Meanwhile, sodium metaborate (NaBO_2_) (from NaBH_4_) behaves as a cross-linking agent between GG molecular chains. The AgNPs/GG hydrogels with excellent viscoelastic properties can be obtained within 3 min at room temperature without the addition of other cross-linkers. The resultant AgNPs/GG hydrogels are flowable and injectable, and they possess excellent pH/thermal responsive properties. Additionally, they exhibit rapid self-healing capacity. This work introduces a facile and scale-up way to prepare a class of hydrogels that can have great potential to biomedical and other industrial applications.

Smart high performance materials, such as stimuli-responsive and self-healing materials have drawn much attention because of their promising applications in a wide range of fields^1^,^2^,^3^,^4^. Hydrogels are a class of promising soft materials possessing high water content and tunable physical properties^5^,^6^,^7^. Recently, a new generation of smart hydrogels possessing stimuli-responsive and self-healing abilities has been suggested^1^,^8^,^9^,^10^,^11^. These hydrogels are regarded as promising materials, especially for biomedical applications including tissue engineering and drug delivery^8^,^12^. However, the present self-healing hydrogels still have some limitations, such as slow self-healing speed or requirements for costly, and poorly industrial-scale synthesis of macromolecular components or complicated chemical modification. Moreover, due to the increasing environmental issues, products based on natural polymers will be desirable.

Metal nanoparticles exhibit size- and shape-dependent properties that endow them with various promising applications in many areas including catalysis, nanoelectronics, nanometal inks and antibiotics^13^,^14^,^15^,^16^. Among these metal nanoparticles, silver nanoparticles (AgNPs) have attracted much attention due to their broad spectrum of antibacterial activity and they are being incrementally applied in medical areas like wound dressings^17^. In addition to their antibacterial activity, AgNPs also possess other features that make them good candidates for molecular rulers^18^ and biosensors^19^. Incorporation of AgNPs into hydrogels can endow hydrogels with enhanced performance and new properties^20^. Furthermore, the three-dimensional hydrogel networks can facilitate the dispersion and stabilization of AgNPs^21^,^22^. Abdel-Halim and his coworker adopted guar gum together with poly(acrylic acid) to prepare hydrogels which were used as a matrix for AgNPs^23^.

Inspired by *in situ* cross-linking approaches^24^,^25^,^26^, we fabricated stimuli-responsive and self-healing AgNPs-containing hydrogels in a simple, mild and scalable method based solely on GG-sodium borohydride (NaBH_4_) system. NaBH_4_ acted as a dual-function agent in this system. Specifically, NaBH_4_ was the reductant for AgNPs synthesis from silver nitrate (AgNO_3_) as the precursor, and sodium metaborate (NaBO_2_), formed from NaBH_4_, was the actual cross-linker in this system. GG was subsequently *in-situ* crosslinked by sodium metaborate, giving rise to hydrogels. The process was extremely fast, which was completed in a matter of a few minutes. The resultant AgNPs/GG hydrogels showed excellent properties including self-healing, pH/thermal responsive and injectable properties. In this way, GG-based hybrid hydrogels containing AgNPs can be readily fabricated in a one-pot process.

## Results

### AgNPs/GG Hydrogels

**Figure 1:**
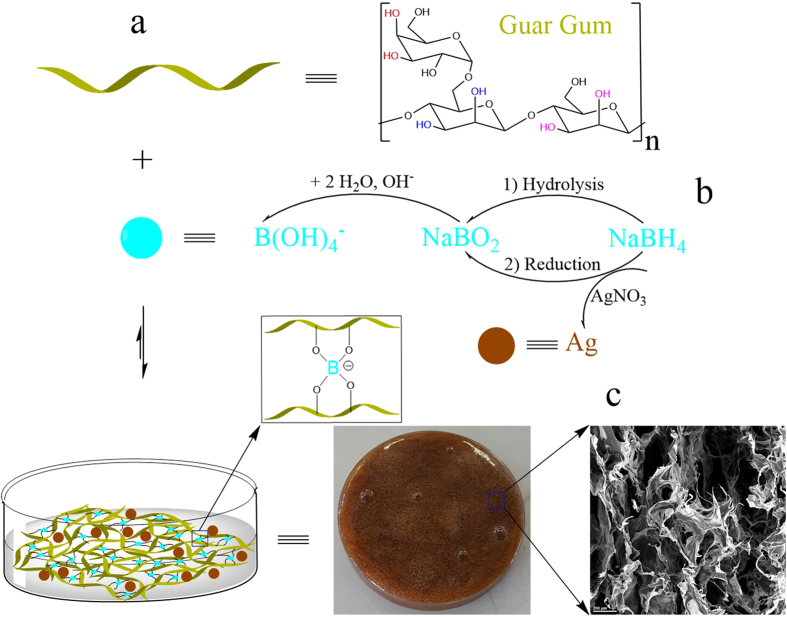
Preparation of AgNPs/GG hydrogels. (**a**) Schematic illustration of AgNPs/GG hydrogels crosslinked by NaBO_2_. (**b**) The generation of NaBO_2_ and AgNPs. (**c**) SEM of AgNPs/GG hydrogels showing their porous structure.

Facile preparation of AgNPs/GG hydrogels was realized by the addition of 3 mL NaBH_4_ (0.1 mol/L) into 50 mL GG aqueous solution (1%, w/v) containing 10 mL of 0.01 mol/L silver nitrate (AgNO_3_). Before the addition of NaBH_4_, the GG/AgNO_3_ solution appeared clear. Upon the addition of NaBH_4_, the color quickly changed to brown, in addition, the viscosity of the solution increased significantly and quickly a brown colored hydrogel formed (Supplementary Video 1). Figure 1 shows the schematic of AgNPs/GG hydrogels formation. Ag^+^ can be reduced to Ag^0^ by NaBH_4_ which also facilitates instant silver nuclei generation. Sodium metaborate (NaBO_2_) from NaBH_4_ in the present system can be a cross-linker for the hydrogel preparation. Specifically, the cis-diol groups on the GG molecules can complex with borate ions that are derived from NaBO_2_. As a result, two cis-diol pairs on adjacent GG molecules can be connected by a borate ion resulting in an inter-molecular cross-linking. Schultz and Myers^27^ used a commercial sodium metaborate to prepare polyvinyl alcohol (PVA) gels and studied the chemorheology of the resultant gels.

We repeated the GG hydrogel fabrication experiment with the addition of NaBH_4_ but in the absence of AgNO_3_ (Supplementary Video 2). The results indicated that the hydrogels could still be obtained quickly. In addition, a set of experiments were implemented to show the existence of NaBO_2_ on GG hydrogels gelation. Particularly, the pH of both NaBH_4_ and GG aqueous solution was pre-adjusted to 13 with sodium hydroxide (NaOH) to hinder the hydrolysis of NaBH_4_, and it turned out that the gelation of GG did not happen even after stirring for 30 min (Supplementary Fig. S1). On the other hand, the NaBH_4_ solution was left for two days to be fully hydrolyzed, which was then used to induce GG cross-linking, forming hydrogels just in a few seconds (Supplementary Video 3). Thus, NaBO_2_, from NaBH_4_, played a critical role in the GG hydrogel formation.

**Figure 2:**
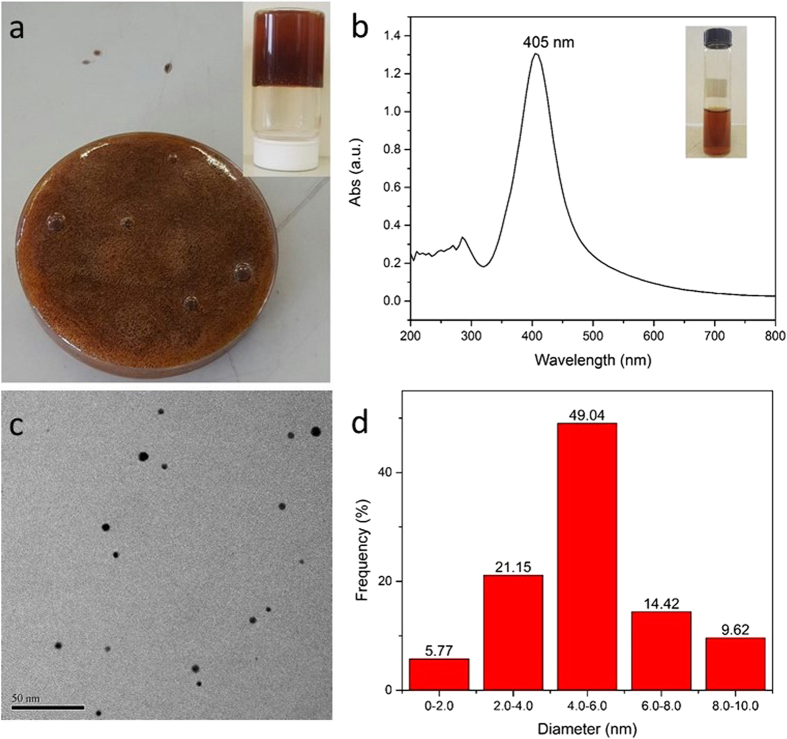
AgNPs/GG hydrogels. (**a**) Photographs of resultant AgNPs/GG hydrogels. **(b)** Ultraviolet-visible spectrum of AgNPs/GG hydrogels. **(c)** TEM of the AgNPs in the GG matrix. (**d**) AgNPs size distributions.

Figure 2 shows the obtained brown colored AgNPs/GG hybrid hydrogels and the AgNPs. Ultraviolet-visible (UV-vis) absorption spectrum (Fig. 2b) and the results on the energy dispersive X-ray (EDX) (Supplementary Fig. S2) were adopted to confirm the formation of AgNPs. As shown in Fig. 2b, a distinct adsorption at 405 nm was evident, which is indicative of AgNPs. Results from EDX analysis provided the direct evidence for the generation of AgNPs. Transmission electron microscopy (TEM) was also used to characterize the formed AgNPs (Fig. 2c), and larger area is shown in Supplementary Fig. S3a. Furthermore, the particle size and the size distribution were also determined (Fig. 2d). The TEM image indicates the well distributed AgNPs within the hydrogels without agglomeration. Furthermore, as shown in the TEM, the AgNPs were spherical, in the range of 2–9 nm. The same AgNPs/GG hydrogel was characterized again after two weeks, which indicated an excellent stability of the AgNPs during the storage (Supplementary Fig. S3b). These results supported the conclusion that GG is a superior matrix for the formed AgNPs. The obtained AgNPs/GG hydrogels were further dried through solvent exchange with ethanol. SEM characterization was adopted to observe the inner structure of the hydrogel (Supplementary Fig. S4) and the results indicated that the hydrogels had rich pores inside.

### Multi stimuli-responsive properties

**Figure 3:**
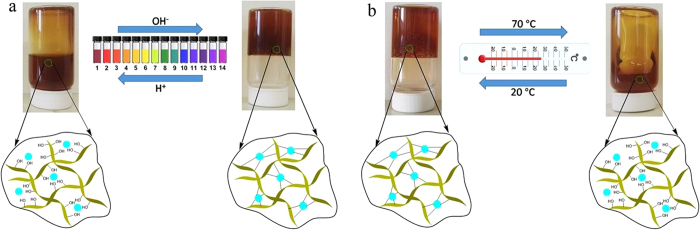
Stimuli-responsive sol-gel transition of AgNPs/GG hydrogels. (**a**) pH responsive sol-gel transition: adding HCl aq. to the hydrogel yielded the sol state, while the addition of NaOH aq. to the sol restored the hydrogel. (**b**) Thermal responsive sol-gel transition: heating the hydrogel to 70 °C yielded the sol state, while the subsequent cooling to 20 °C restored the hydrogel.

Since borates and cis-diols interactions are versatile and reversible, we believe that it can be used to prepare intelligent functional hydrogels with stimuli-responsive properties. In particular, because of the reversible linkages between GG hydroxyl groups and B(OH)_4_^−^, the obtained GG-based hydrogels can respond to both pH and temperature variations. To eliminate the influence of AgNPs, we firstly tested the stimuli responsive performance of GG hydrogels without AgNPs. The results are shown in Supplementary Fig. S5. GG hydrogels prepared in this method clearly exhibited the multi stimuli-responsive properties. For AgNPs containing GG hydrogels, as shown in Fig. 3a, they can be disintegrated into a viscous solution when a HCl solution was added. In contrast, the hydrogels were recovered when a NaOH solution was added. This can be attributed to the dynamic transition between B(OH)_4_^−^ and B(OH)_3_ upon the pH variation: B(OH)_4_^−^ is an effective crosslinking agent for GG, while B(OH)_3_ is not; B(OH)_4_^−^ was converted to B(OH)_3_ upon the addition of a HCl solution, causing the disintegration of hydrogels; conversely, with the addition of NaOH, B(OH)_4_^−^ was formed, thus, the hydrogels were recovered. Lu *et al.*^28^ reported in their study of hydroxypropyl guar (HPG)-borate interactions that the interactions between HPG and borate was sensitive to pH. The sol-gel transition also occurred during the heating/cooling process: the hydrogel became liquid when heated to 70 °C, while the hydrogel was re-formed after cooled down to 20 °C (Fig. 3b). This thermal response was attributed to reversible, exothermic reactions between B(OH)_4_^−^ and GG hydroxyl groups. Schultz and Myers^27^ also reported the reversible, exothermic reactions between borate ion and polyols. Therefore, it can be concluded from the results that AgNPs/GG hybrid hydrogels also exhibited their stimuli-responsive properties due to pH/thermal changes indicating that the incorporation of AgNPs did not affect the interactions between B(OH)_4_^−^ and GG hydroxyl groups.

### Self-healing and injectable properties

**Figure 4:**
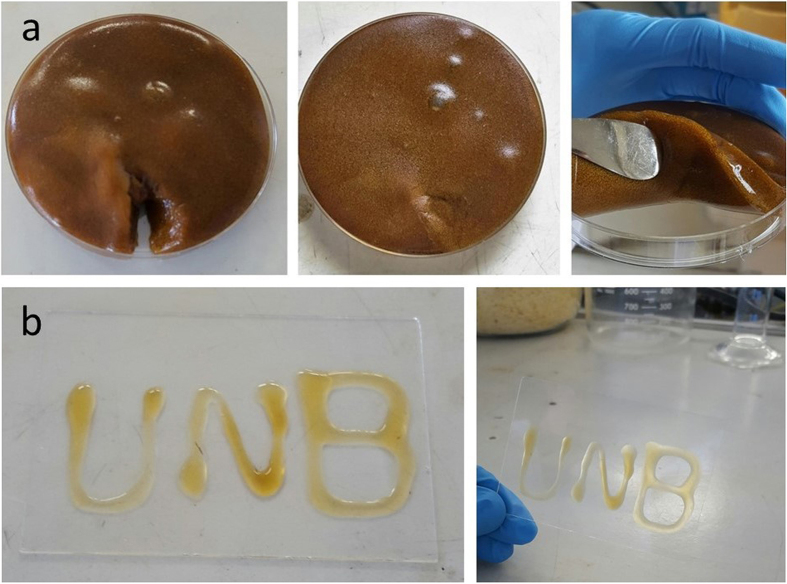
Self-healing and injectable tests. (**a**) AgNPs/GG hydrogels recovered from a cut without external interventions under room temperature; (**b**) AgNPs/GG hydrogels were extruded through a syringe directly onto a glass slide, which showed their injectable properties (the AgNPs/GG hydrogels used for injectable testing were prepared using 0.5% (w/v) GG solution).

The obtained GG hydrogels without AgNPs were firstly taken to study their self-healing and injectable properties (Supplementary Fig. S6). The results certificated that GG hydrogels had rapid and excellent self-healing ability due to the existing dynamic networks between GG hydroxyl groups and B(OH)_4_^−^ (Supplementary Video 4 shows the self-healing process). A bunch of research was also carried out to test the self-healing and injectable properties of the resultant AgNPs/GG hybrid hydrogels. Specifically, as depicted in Fig. 4a, a hydrogel was sliced and one small portion was removed, then very quickly the hydrogel recovered the cut without external interventions under room temperature. This result proved the excellent self-healing performance of the AgNPs containing product.

After the gelation, AgNPs/GG hybrid hydrogels were extruded through a 21-gauge needle directly onto a glass slide to test their injectable properties. As shown in Fig. 4b, the obtained AgNPs/GG hybrid hydrogels were syringe-injectable. They can firmly stick to the glass slide without falling or sliding off even when the glass slide was vertically placed. This injectable property can be ascribed to an equilibrium: enough shear force will break the gel, and it can re-form quickly once the shear force is gone. The injectable characteristics can be significant to biomedical applications, for instance, it could be potentially used as scaffolds for 3D support of cells. Consequently, all these tests on AgNPs/GG hybrid hydrogels showed the proof of their self-healing and injectable properties, which indicates that the incorporation of AgNPs did not change these properties of GG-based hydrogels either.

## Discussion

For the first time, a new smart AgNPs/GG hydrogel was developed utilizing a GG-NaBH_4_ system, and the resultant hydrogel showed multi stimuli-responsive, self-healing and injectable properties. It was specially noted that the self-healing was very fast. In this system, NaBH_4_ has dual functions: (1) NaBH_4_ is a reducing agent so that silver nanoparticles (AgNPs) were successfully synthesized *in-situ*; (2) NaBO_2_ generated from NaBH_4_, is the cross-linker for GG hydrogels. The sol-gel switching of the obtained AgNPs/GG hydrogels can be realized by changing pH and/or temperature. The fast self-healing capacity under mild conditions (e.g., room temperature) was also demonstrated. Because of the simplicity, high-efficiency, and cost-effectiveness of the preparation process, these AgNPs/GG hydrogels could have a bright future in the biomedical applications. Moreover, this research demonstrates a class of intelligent metal nanoparticles/natural polymer-based hydrogels that can be synthesized in a one-step process using NaBH_4_.

## Methods

### Preparation of guar gum (GG)/silver nanoparticles (AgNPs) hydrogels

5 g GG was progressively added to 500 mL deionized water under constant stirring (300 rpm) to prepare 1% (w/v) GG solution. Then, 50 mL 1% (w/v) GG aqueous solution was transferred to a beaker. Subsequently, 10 mL newly prepared silver nitrate (AgNO_3_, 0.01 mol/L) aqueous solution was added to the GG solution. After stirring for 1 min, a 3 mL NaBH_4_ (0.1 mol/L) solution was subsequently added to the system under stirring, and the color of the mixture changed quickly to brown, the hydrogel was formed.

### Preparation of guar gum (GG) hydrogels

50 mL 1% (w/v) GG aqueous solution was transferred to a beaker. A 3 mL 0.1 mol/L sodium borohydride (NaBH_4_) aqueous solution freshly prepared was added dropwise to a beaker containing the GG solution under stirring. The GG-based hydrogel was quickly formed upon the addition of NaBH_4_.

### Characterization

For the ultraviolet-visible (UV-vis) analysis, the resultant AgNPs/GG hydrogel sample was dispersed in deionized water by agitation to get a diluted suspension. Then, about 2.5 mL of upper solution was taken for the UV-vis analysis, and the data were collected on a GENESYS 10 UV-Vis spectrophotometer from 200 to 800 nm wavelength (Thermo Fisher Scientific Inc.) using deionized water as the background. The samples for transmission electron microscopy (TEM) observation were also prepared by disintegrating the hydrogel in water. A drop of the aforementioned suspension was cast on a carbon-coated copper grid for TEM observation. The TEM study was carried out on a JEOL 2011 with a 200 kV acceleration voltage. The size and size distribution of AgNPs were measured and analyzed using an ImageJ software. The elemental analysis of the hydrogel samples was carried out with an energy dispersive X-ray system (EDX) attached to the TEM. For the scanning electron microscopy (SEM) observation, the resultant hydrogels were dried based on the solvent exchange technique using ethanol. The samples were treated/fractured in liquid nitrogen for the cross-section observation. SEM images were taken using a JEOL 6400 microscopy with a 15 kV acceleration voltage.

## Additional Information

**How to cite this article**: Dai, L. *et al.* Silver nanoparticles-containing dual-function hydrogels based on a guar gum-sodium borohydride system. *Sci. Rep.* **6**, 36497; doi: 10.1038/srep36497 (2016).

**Publisher’s note:** Springer Nature remains neutral with regard to jurisdictional claims in published maps and institutional affiliations.

## Supplementary Material

Supplementary Information

Supplementary Video 1

Supplementary Video 2

Supplementary Video 3

Supplementary Video 4
