# Supplementary material for: Silver nanoparticles-containing dual-function hydrogels based on a guar gum-sodium borohydride system
Source: Sci Rep. 2016 Nov 7;6:36497. doi: 10.1038/srep36497 (PMC5098157; doi:10.1038/srep36497)
Supplement: Supplementary Information [file srep36497-s1.pdf]

Supplementary information

Silver nanoparticles-containing dual-function hydrogels based on a guar gum-sodium borohydride system

Lei Dai,[1] Ben Nadeau,[1] Xingye An,[1] Dong Cheng,[1] Zhu Long,[2] & Yonghao Ni*[1]

1. Department of Chemical Engineering, University of New Brunswick, Fredericton, NB E3B 5A3, Canada

2. Key Laboratory of Eco-textiles, Ministry of Education, Jiangnan University, Wuxi 214122, China

Correspondence and requests for materials should be addressed to Y. N. (e-mail: yonghao@unb.ca)

Supplementary Figures

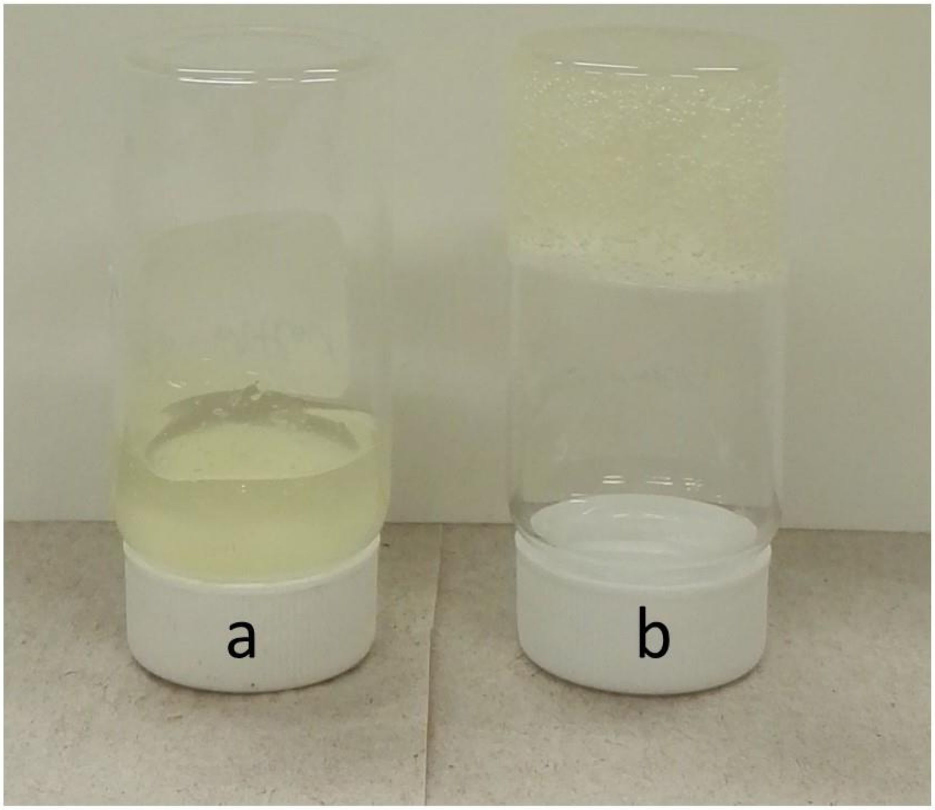

Supplementary Figure S1. Experimental in demonstrating the role of $NaBO_2$ for the formation of GG hydrogels. **(a)** The GG solution (pH = 13.0 ± 0.1) was mixed with $NaBH_4$ aq. (pH = 13.0 ± 0.1), under the conditions, no $NaBO_2$ was formed, thus no gelation occurred. **(b)** the GG solution was mixed with fresh prepared $NaBH_4$ aq. (pH = 9.6 ± 0.1), under the conditions, $NaBO_2$ was formed due to hydrolysis, thus gelation occurred.

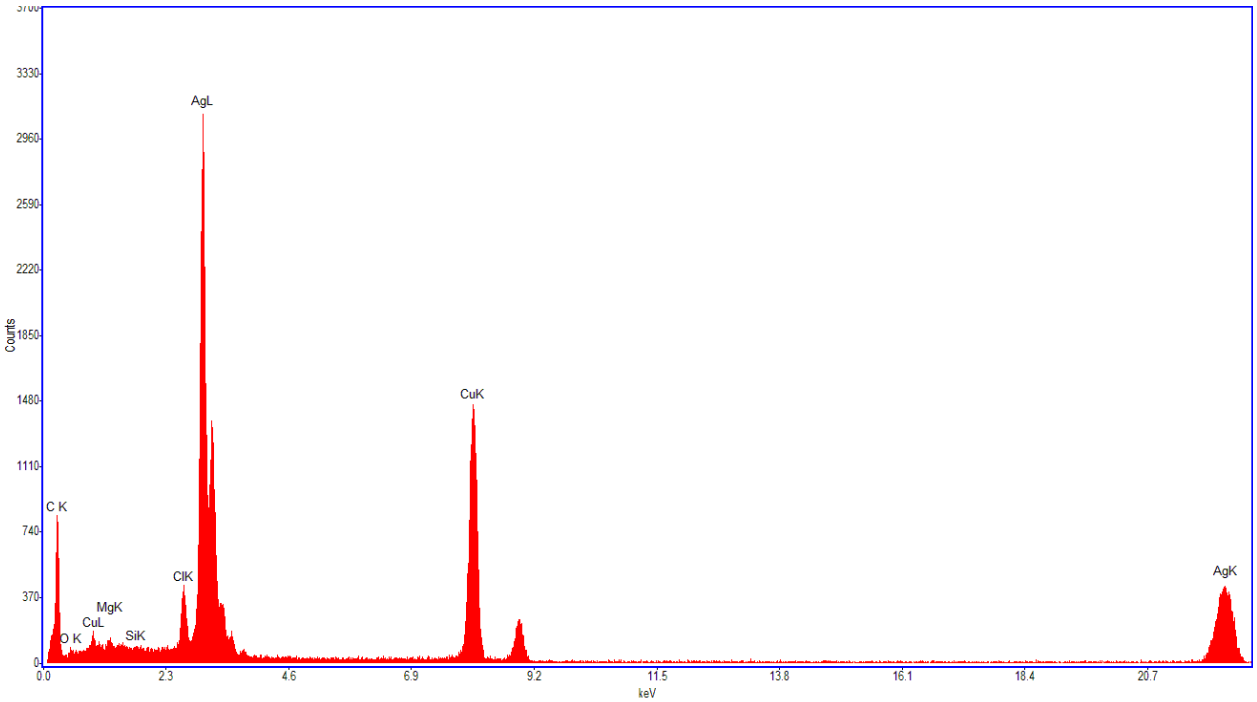

Supplementary Figure S2. EDX analysis of the AgNPs/GG hydrogels, providing the direct evidence for the generation of AgNPs.

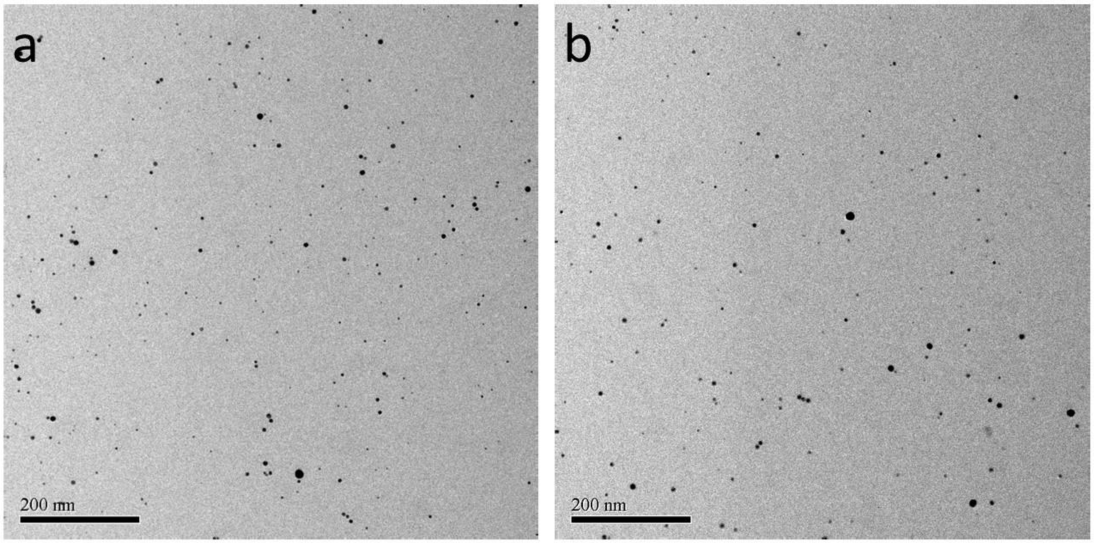

Supplementary Figure S3. TEM pictures of AgNPs in GG-based hydrogels. **(a)** Freshly prepared sample. **(b)** The sample stored for two weeks (it showed that the AgNPs were stable in the hydrogel).

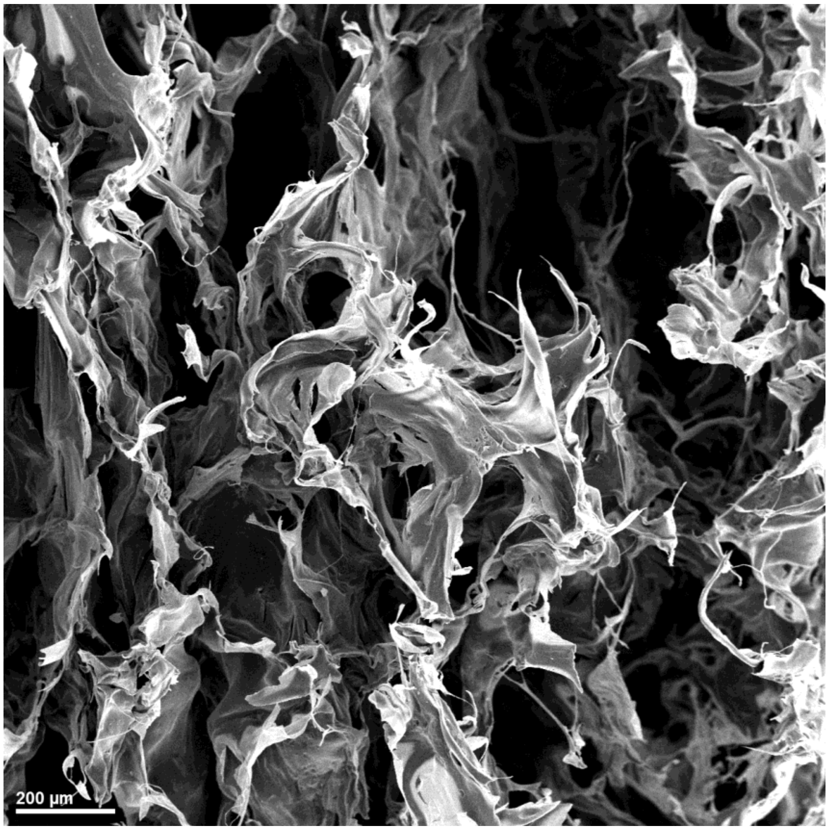

Supplementary Figure S4. SEM picture of cross-section of dried AgNPs/GG hydrogels, showing the porous structure of the hydrogels.

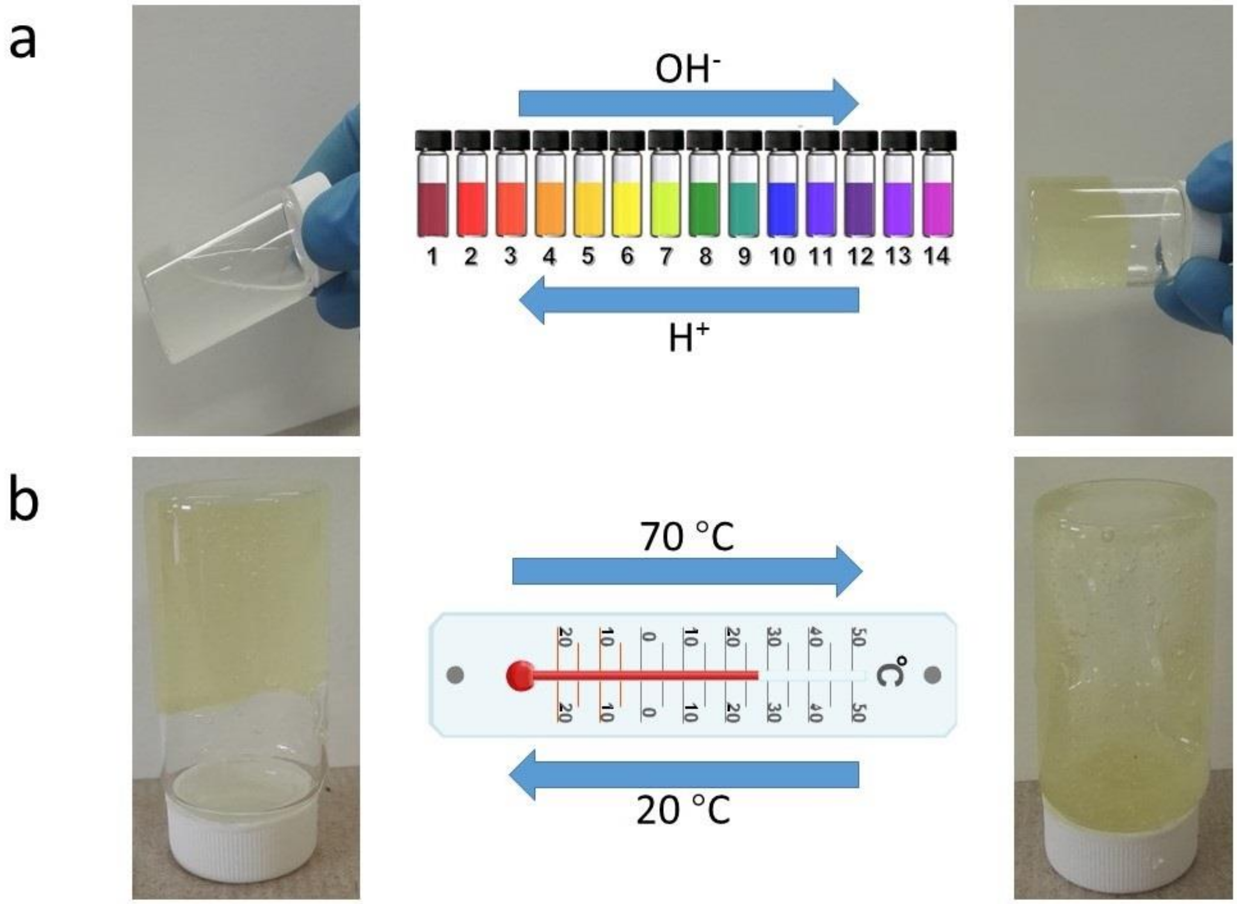

Supplementary Figure S5. Multi-stimuli responsive properties of GG-based hydrogels. **(a)** pH responsive performance of GG hydrogels (the sol-gel switch can be realized by pH changes). **(b)** Thermal responsive performance of GG hydrogels (the sol-gel switch can be realized by temperature changes).

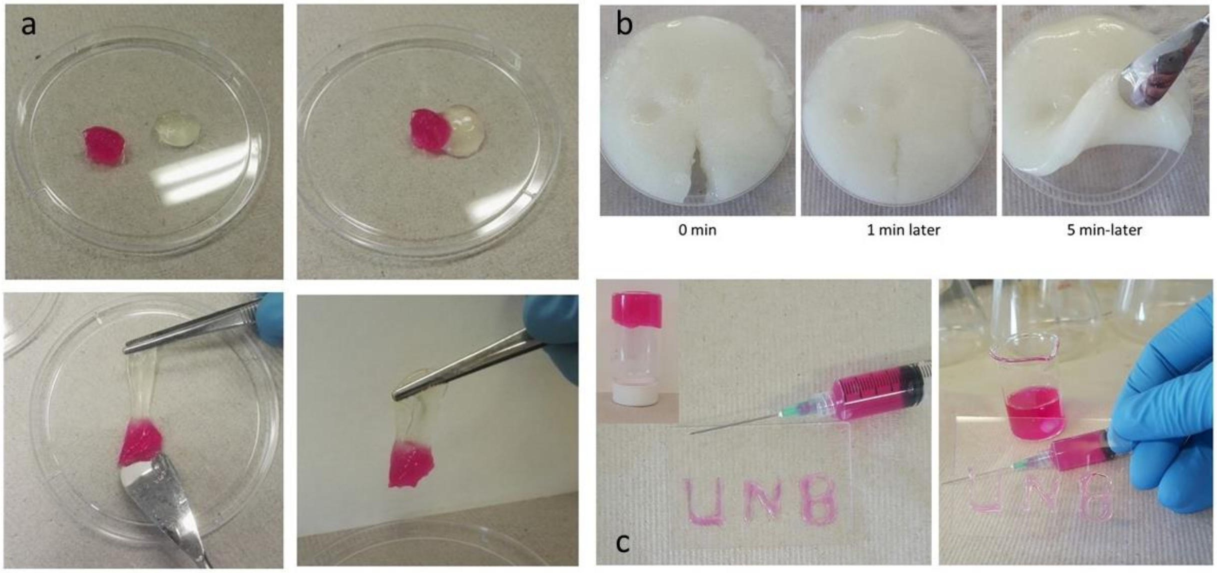

Supplementary Figure S6. Self-healing and injectable properties of GG-based hydrogels**. (a)** Two pieces of hydrogels (one of which was dyed to red to show the healing between the two) merged to a homogeneous one. **(b)** The hydrogel recovered from the cutting wound. **(c)** Hydrogels were injected by a syringe onto a glass slide (the hydrogels for injectable testing were prepared using 0.5% (w/v) GG solution).

Supplementary Video 1. Preparation of AgNPs/GG hydrogels using freshly prepared $NaBH_4$

Supplementary Video 2. Preparation of GG hydrogels using freshly prepared $NaBH_4$

Supplementary Video 3. Preparation of GG hydrogels using fully hydrolyzed $NaBH_4$

Supplementary Video 4. Self-healing properties of GG hydrogels
